# Commentary: Raw and Cooked Vegetable Consumption and Risk of Cardiovascular Disease: A Study of 400,000 Adults in UK Biobank

**DOI:** 10.3389/fnut.2022.896500

**Published:** 2022-05-16

**Authors:** David E. Most

**Affiliations:** School of Education, Colorado State University, Fort Collins, CO, United States

**Keywords:** vegetable intake, cardiovascular mortality, scientific inference and reasoning, statistical inference abuse, meta-analytic thinking

## Introduction

The purpose of this commentary is to offer a constructive critique on one of the principal findings of this important and interesting study. As described, the objective of the study is to learn more about the independent effects of raw and cooked vegetable consumption on cardiovascular disease (CVD) (1). Associations between vegetable intake and two primary outcomes, namely CVD incidence and CVD mortality, are modeled, and the key quantities of interest are adjusted hazard ratios (HR). The claim is made that cooked vegetable intake and raw vegetable intake showed different associations with both cardiovascular outcomes. In the Discussion section (1), Feng et al. write “When assessing the independent effect of raw and cooked vegetable intake, only raw vegetable intake showed inverse associations with CVD outcomes, whereas cooked vegetables showed no association.” The problem with this claim is that, in the case of one of the two primary outcomes, CVD mortality, the evidence does not seem to support such a conclusion.

## Evidence and Interpretation

What does the evidence seem to indicate? Figure 3 presents the HR estimates of the relationship between the level of vegetable intake, relative to the lowest consumption level, and CVD mortality, along with 95% CIs, from the fully adjusted models (1). For both raw vegetables and cooked vegetables, the levels of intake were categorized into four levels (tablespoons/day): 0, 1–2, 3–4, and >4. Using zero as the reference category, the HR estimates for raw vegetables for the three categories above the reference category are 0.89, 0.92, and 0.85. The equivalent HR estimates for cooked vegetables are 0.88, 0.87, and 0.96. On its face, an examination of these HR estimates suggests that raw vegetables and cooked vegetables have similar relationships with CVD mortality. In other words, if it is reasonable here to draw a substantive conclusion that raw vegetable intake showed an inverse association with CVD mortality, then the same ought to be said for cooked vegetables.

Why is there a discrepancy between the evidence offered in Figure 3 and the prose characterization of the results? The reason for the discrepancy is a common error in interpretation. The mistake is to conflate a binary statistical declaration with a substantive conclusion. In particular, a declaration of no statistically significant association is conflated with a scientific conclusion that no evidence was found for an association or simply of “no association.” The interpretation of the results, as presented, is based on a binary declaration regarding statistical significance, or, equivalently, whether a 95% CI for the HR includes one, rather than on an evaluation of the magnitude of the HR estimates. Feng et al. happen to focus on comparing the highest (>4) vs. the lowest level of vegetable intake, though the aforementioned inappropriate conflation would yield similar interpretational errors if considering other levels of vegetable intake. In short, we shouldn't conclude that there is no association because of a binary statistical decision (e.g., *p* > 0.05, 95% CI for HR includes one), and we should not conclude that two results are different because of differences in statistical significance (2).

What about uncertainty in the HR estimates? The presentation of 95% CIs in Figure 3 is helpful for quantifying uncertainty in HR estimates. Across all levels of intake for both raw and cooked vegetables, the plausible true values of reduction in risk in CVD mortality, compared to the reference category, range from a high of 20–25% to something close to zero. In other words, if uncertainty is taken into account, the difference in ranges of plausible true HR values that are compatible with the data seem clinically indistinguishable when comparing raw and cooked vegetable intake. Embracing this uncertainty in HR estimates further supports the notion that the evidence is not consistent with an interpretation that raw and cooked vegetables showed different associations with CVD mortality.

How do the findings align with previous studies? Given that both raw vegetable intake and cooked vegetable intake are associated with a reduced risk of CVD mortality (and all-cause mortality), it seems that the findings are entirely consistent with the EPIC study, in this regard. The two other identified prior studies examined the relationship between vegetable intake by type and all-cause mortality. In the case of reviewing the Prospective Urban Rural Epidemiology (PURE) study, Feng et al. mischaracterize the findings. In the Introduction, Discussion, and Table 10, they describe the PURE study as having found an inverse association with all-cause mortality for raw vegetable intake, but not for cooked vegetable intake (1). However, the PURE paper explicitly says that “In the fully adjusted models, both raw and cooked vegetable intakes were inversely associated with total mortality” (3). In the case of reviewing the Australian cohort study, Feng et al. again conflate a statistical declaration with a substantive conclusion, when they write that “only cooked vegetable intake was associated with lower overall mortality” (1). In the Results section of the Australian cohort study paper, the authors note that “The association with raw vegetable consumption showed estimates (and CIs) that were consistent with those for cooked vegetables” (4). Examination of Table 2 suggests that this clinical interpretation is reasonable. Therefore, from a meta-analytic perspective, the findings with regard to all-cause mortality are actually consistent across all four studies.

## Discussion

The concern described here might be considered an example of a more general century-old problem of not distinguishing between statistical inference and scientific inference (5). Empirical examinations of the literature in various disciplines suggest that associated interpretational errors happen more often than not (2). For example, the PURE study authors prominently make this sort of mistake in their “Interpretation,” as they write that “Higher fruit, vegetable, and legume consumption were associated with a lower risk of non-cardiovascular, and total mortality,” explicitly excluding the association with CVD mortality, despite the fact that it was with CVD mortality that the highest reduction in risk was observed (3). While statistics offers useful tools for quantifying some types of uncertainty, generating cumulative knowledge depends on summaries of findings that have fidelity to the evidence.

## Author Contributions

The author confirms being the sole contributor of this work and has approved it for publication.

## Conflict of Interest

The author declares that the research was conducted in the absence of any commercial or financial relationships that could be construed as a potential conflict of interest.

## Publisher's Note

All claims expressed in this article are solely those of the authors and do not necessarily represent those of their affiliated organizations, or those of the publisher, the editors and the reviewers. Any product that may be evaluated in this article, or claim that may be made by its manufacturer, is not guaranteed or endorsed by the publisher.
